# Ionizing radiation induces tumor cell lysyl oxidase secretion

**DOI:** 10.1186/1471-2407-14-532

**Published:** 2014-07-22

**Authors:** Colette J Shen, Ashish Sharma, Dinh-Van Vuong, Janine T Erler, Martin Pruschy, Angela Broggini-Tenzer

**Affiliations:** 1 Laboratory for Molecular Radiobiology, University Hospital Zurich, 8091 Zürich, Switzerland; 2 Raymond and Ruth Perelman School of Medicine, University of Pennsylvania, 19104 Philadelphia, PA, USA; 3 Biotech Research & Innovation Centre (BRIC), University of Copenhagen, 2200 Copenhagen, Denmark

**Keywords:** Lysyl oxidase, Ionizing radiation, Tumor invasion, Radiation resistance, Hypoxia, Microtubule stabilizing agent

## Abstract

**Background:**

Ionizing radiation (IR) is a mainstay of cancer therapy, but irradiation can at times also lead to stress responses, which counteract IR-induced cytotoxicity. IR also triggers cellular secretion of vascular endothelial growth factor, transforming growth factor β and matrix metalloproteinases, among others, to promote tumor progression. Lysyl oxidase is known to play an important role in hypoxia-dependent cancer cell dissemination and metastasis. Here, we investigated the effects of IR on the expression and secretion of lysyl oxidase (LOX) from tumor cells.

**Methods:**

LOX-secretion along with enzymatic activity was investigated in multiple tumor cell lines in response to irradiation. Transwell migration assays were performed to evaluate invasive capacity of naïve tumor cells in response to IR-induced LOX. *In vivo* studies for confirming IR-enhanced LOX were performed employing immunohistochemistry of tumor tissues and *ex vivo* analysis of murine blood serum derived from locally irradiated A549-derived tumor xenografts.

**Results:**

LOX was secreted in a dose dependent way from several tumor cell lines in response to irradiation. IR did not increase LOX-transcription but induced LOX-secretion. LOX-secretion could not be prevented by the microtubule stabilizing agent patupilone. In contrast, hypoxia induced LOX-transcription, and interestingly, hypoxia-dependent LOX-secretion could be counteracted by patupilone. Conditioned media from irradiated tumor cells promoted invasiveness of naïve tumor cells, while conditioned media from irradiated, LOX- siRNA-silenced cells did not stimulate their invasive capacity. Locally applied irradiation to tumor xenografts also increased LOX-secretion *in vivo* and resulted in enhanced LOX-levels in the murine blood serum.

**Conclusions:**

These results indicate a differential regulation of LOX-expression and secretion in response to IR and hypoxia, and suggest that LOX may contribute towards an IR-induced migratory phenotype in sublethally-irradiated tumor cells and tumor progression.

## Background

Ionizing radiation (IR) is a mainstay of cancer therapy, but resistance to therapy often leads to recurrence and poor outcome for cancer patients. Several studies have uncovered sources of intrinsic resistance to radiation, such as hypoxia, activation of oncogenes and cell signaling pathways and defects in apoptosis and DNA damage repair [1-6]. However, mechanisms of acquired resistance to IR are poorly understood. Several studies have suggested that radiation can lead to stress responses that allow continued tumor survival and progression, thus hindering its therapeutic benefit. For instance, IR stimulates the activation of the hypoxia-inducible transcription factor (HIF)-1 and subsequent expression and secretion of the tumor vascular endothelial growth factor (VEGF) to protect the tumor vasculature [7,8]. IR also induces expression of the transforming growth factor β (TGF-β) and thereby TGF-β-mediated epithelial-to-mesenchymal transition in mammary epithelial cells [9], and activation of the epidermal growth factor receptor (EGFR) thereby promoting tumor growth [10]. Further, IR-induced matrix metalloproteinase activity enhances the invasive capacity of multiple cancer cell types, including melanoma, prostate cancer, pancreatic cancer, hepatocellular carcinoma and glioma [11-17] and initiates myeloid cell recruitment relevant for vasculogenesis [18,19]. IR also upregulates integrin expression promoting a cellular invasive phenotype [17,20].

Lysyl oxidase (LOX) is a cell-secreted amine oxidase that crosslinks collagen and elastin in the extracellular space, resulting in increased tissue stiffness and tensile strength. It is secreted as an inactive 50 kDa proenzyme and cleaved extracellularly to a 32 kDa active enzyme [21,22]. To date, LOX has been shown to be induced by hypoxia and several cytokines, including TGF-β [23-25]. While its role in the morphogenesis and repair of connective tissues is well established, recent work has suggested an important role in cancer progression [26]. High tumor LOX expression is associated with poor distant metastasis-free and overall survival in patients. Furthermore, LOX promotes tumor growth and progression *in vivo*, cancer cell invasion, and premetastatic niche formation to foster distant metastases [22,27-30]. As part of an autocrine loop LOX also drives VEGF-expression and subsequent tumor angiogenesis through LOX-activated PDGF-receptor signaling [31]. In this study, we investigate the effects of IR on LOX secretion by tumor cells to determine a putative role in an IR-induced stress response, which might promote treatment resistance. We demonstrate that clinically relevant doses of IR enhance LOX secretion *in vitro* and *in vivo* and that IR-induced LOX stimulates tumor cell invasion on a functional level. Furthermore, our expression and combined treatment studies with microtubule-stabilizing agents suggest that LOX expression and secretion are differentially regulated by hypoxia and ionizing radiation.

## Methods

### Cell culture, reagents, and irradiation

All cell culture media and supplements were obtained from Gibco (Invitrogen). The human lung adenocarcinoma cells A549 were grown in RPMI 1640 medium, and the human colon adenocarcinoma cells SW620 were grown in DMEM. All media were supplemented with 10% (v/v) fetal bovine serum, 1% (v/v) penicillin-streptomycin, and 1% (v/v) L-glutamine, and cells were cultured at 37°C in a 5% CO_2_ humidified incubator. Additional cell lines used for the exhaustive screening included H125 (lung cancer); HCT116, HT29, SW480 (colon cancer); LN18, U251 (glioma); D341, DAOY (medulloblastoma); A431 (vulval cancer) and MDA-MB-231 (breast cancer). These cell lines were also grown in the fully supplemented RPMI1640-medium.

Patupilone (Epothilone B, EPO906) was provided by Novartis Pharma AG (Basel, Switzerland). To prepare conditioned cell culture medium (CM), cells were initially seeded in serum-containing medium for 24-48 hours and then sham-treated or irradiated with the indicated doses of IR. The cell culture medium was discarded 1 hour later, and cells were rinsed once in PBS and incubated for an additional 16-20 hours in exactly 10 ml of serum-free culture medium. Conditioned cell culture medium was collected and immediately filtered through a 0.45 μm sterile filter to remove any floating cells, then concentrated in 10,000 NMWL Centricon filter devices (Millipore) by centrifuging at 4000 × g for 15 minutes at 4°C to exactly 400 μl of concentrated CM. The total amount of protein in concentrated CM was determined using a NanoDrop Spectrophotometer. Concentrated CM was stored at -80°C. For patupilone treatment, cells were pretreated with DMSO (control) or 0.5 nM patupilone 24 hours prior to irradiation. Irradiation was performed at room temperature using a Primart 6 MV X-ray linear accelerator unit (Siemens) at 2.8 Gy/min or an Xstrahl 200 kV X-ray unit at 1 Gy/min.

### siRNA transfection

Transfection was performed using backward transfection with Lipofectamine RNAiMAX (Invitrogen). siRNAs for downregulation of human LOX (NM_002317.5) and firefly luciferase (control) were synthesized by Microsynth (Switzerland) and used at 20 nM concentration. siRNA sequences are as follows (5’-3’): siLOX, CAAUGCUCCUACUGUUUAAdTdT; siLuc, CGUACGCGGAAUACUUCGAdTdT.

### Quantitative real-time RT-PCR

Sample RNA was isolated using an RNeasy Mini Kit (Qiagen), then quantified using a NanoDrop spectrophotometer. RNA was reverse-transcribed using a High Capacity cDNA Reverse Transcription Kit (Applied Biosystems), and the resulting cDNA was amplified using FastStart Universal SYBR Green Master Mix (Roche) on an Applied Biosystems 7900HT real-time PCR instrument. Gene expression values represent 2^-ΔΔCt^, normalized to 18S rRNA. The following primers were used (5’-3’): LOX forward, CGTCCACGTACGTGCAGAAG; LOX reverse, CCTGTATGCTGTACTGGCCAGAC; CDKN1A forward, GGACCTGGAGACTCTCA; CDKN1A reverse, CCTCTTGGAGAAGATCAG; 18S forward, ATGGCCGTTCTTAGTTGGTG; 18S reverse, CGCTGAGCCAGTCAGTGTAG.

### Immunoblotting, antibody array and ELISA

LOX secretion was determined by Western blotting performed on concentrated CM samples using an antibody against LOX (NB100-2527, Novus Biologicals). Angiogenesis array on conditioned media was performed using the Human Angiogenesis array (R&D systems) according to the manufacturer`s protocol. For ELISA, cells derived from different tumor entities were sham-treated or irradiated and conditioned media was harvested 24 h after treatment. LOX protein levels were measured according to the protocol of the commercially available LOX-ELISA-kit (USCN Life Science Inc.). Simultaneous quantification of the number of viable cells was performed to allow correction of LOX -levels for cell number.

### LOX and LDH activity assay

To assess LOX enzymatic activity in CM, a fluorometric assay detecting the level of hydrogen peroxide was performed as previously described [32] using a freshly prepared enzyme mixture containing 1.2 M urea (Sigma-Aldrich), 50 mM sodium borate pH 8.0 (Sigma-Aldrich), 0.01 M calcium chloride (Sigma-Aldrich), and 1.0 U/mL horseradish peroxidase (Sigma-Aldrich), followed by 50 μL of freshly prepared substrate mixture containing 1.2 M urea (Sigma-Aldrich), 50 mM sodium borate pH 8.0 (Sigma-Aldrich), 0.01 M calcium chloride (Sigma-Aldrich), 0.01 M diaminopentane (Sigma-Fluka), and 10 μM amplex red (Invitrogen). To determine lactate dehydrogenase (LDH)-release conditioned media was harvested from sham-treated and irradiated A549 cells, 24 hours after irradiation, LDH-activity was determined using the CytoTox 96 Non-Radioactive Cytotoxicity Assay (Promega). Purified LDH was used as a positive control.

### Transwell invasion assay

To assess cellular invasion in response to experimental manipulations, naïve A549 cells (15,000 cells/insert) were seeded in serum-free medium on Transwell inserts (6.5 mm, 8 μm pores, Costar) coated with 50 μg/ml rat tail collagen I (Becton Dickinson). CM from A549 cells first transfected with control or LOX siRNA and then treated with 0 or 5 Gy IR was used to attract migrating cells. For CM preparation, after irradiation, the same number of cells from each condition was seeded in serum-containing medium, and CM was collected after 24 hours. Cells seeded in inserts were allowed to migrate for 24 hours. For quantification, cells from the upper side of the insert were scraped away with cotton swabs, and then inserts were fixed in 75% methanol/25% acetic acid (v/v) and stained with DAPI. Invaded cells were counted manually under a fluorescent microscope.

### Tumor xenograft experiments and immunohistochemistry

A549 tumor xenograft experiments were carried out as previously described [33]. At a tumor volume of 200-250 mm^3^, tumors were sham-irradiated or irradiated using a customized shielding device with 1 or 2 × 10 Gy, respectively, using an Xstrahl 200 kV X-ray unit at 1 Gy/min. IR-treated animals were sacrificed 24 h after the last fraction of IR-treatment or 48 hours after the last fraction of high dose IR-treatment. Blood serum was derived from heart-punctured animals following euthanasia. For immunohistochemistry tumor xenografts were fixed in 4% PBS-buffered formalin and embedded in paraffin. Sections (3 mm) were mounted on glass slides, deparaffinized, rehydrated and stained with H&E or a LOX-specific antibody (Novus Biologicals, NB100-2527). Whole tumor sections were quantified for specific LOX-staining intensities. Each treatment group consisted of 3 animals and at least 3 sections per tumor were analyzed. All *in vivo* experiments were performed in strict accordance with the guidelines for the welfare and use of animals of the Swiss Cantonal Veterinary Authorities and approved by the same Authorities (Permit Number: 145/2011).

### Statistical analysis

Statistical analysis was performed using Student`s t-test and one- or two-way ANOVA analysis. All experiments were conducted as at least 3 independent times. Results are plotted as mean ± SEM; the level of significance was set at P < 0.05 (*) and P < 0.005 (**).

## Results

### Ionizing radiation promotes LOX-secretion by tumor cells

**Figure 1:**
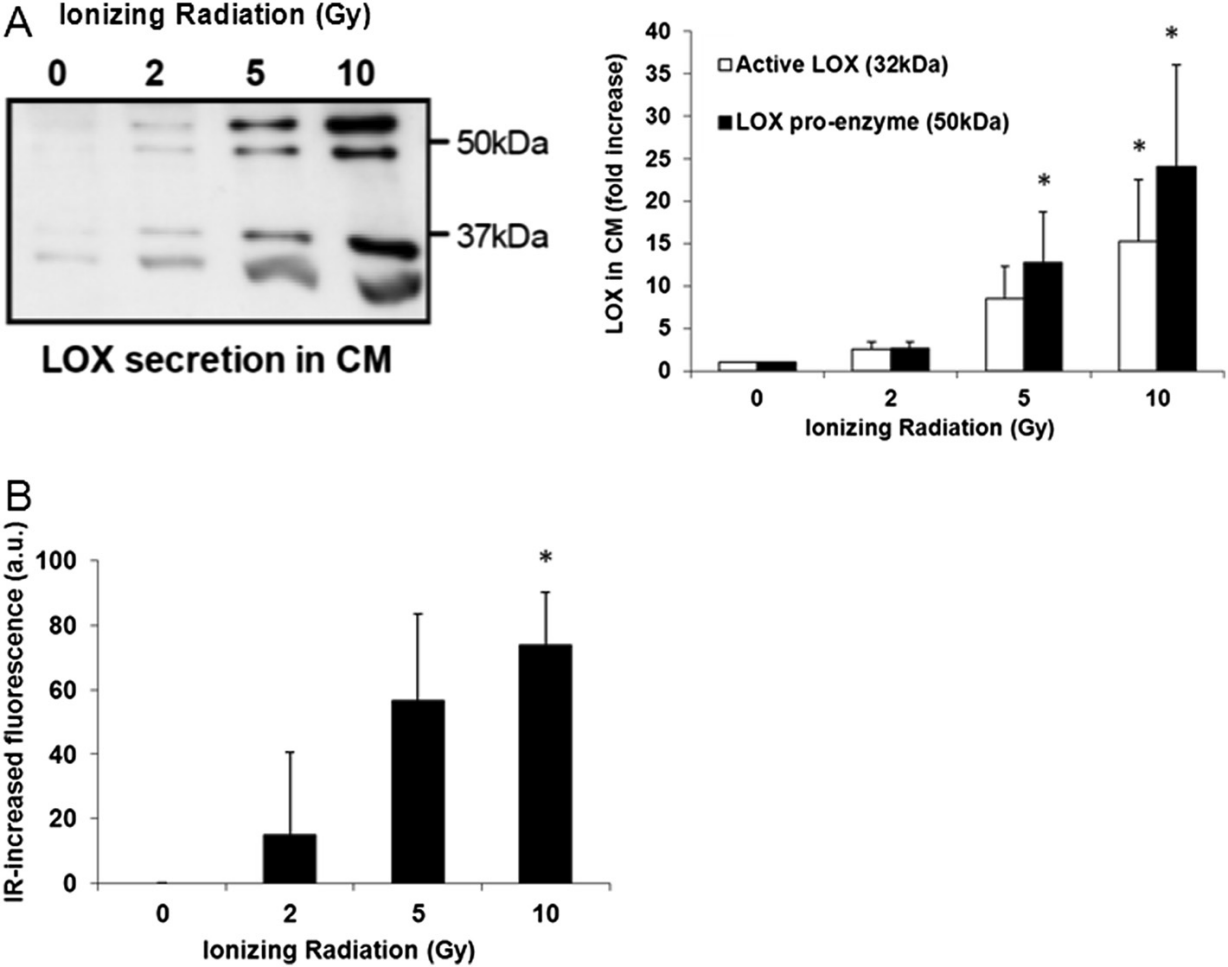
**LOX secretion and activity in response to tumor cell irradiation. (A)** Western blot of LOX zymogen (50 kD) and active form (32 kD) in conditioned media derived from irradiated A549 lung carcinoma cells 16-20 h after irradiation and quantification of band intensities from 3 independent experiments. **(B)** LOX activity in conditioned media derived from irradiated A549 lung carcinoma cells 16-20 h after irradiation, averaged over 3 independent experiments (*: significantly different vs control samples, p < 0.05).

To explore the cellular stress response to ionizing radiation (IR) with regard to LOX, human A459 lung adenocarcinoma cells were irradiated with increasing doses of IR. 16-20 hours after irradiation, the amount and the enzymatic activity of LOX secreted into conditioned medium (CM) were determined by western blotting, ELISA and a fluorometric activity assay, respectively. Interestingly, we noted increased secretion of both active LOX enzyme and inactive LOX pro-enzyme by human A549 lung adenocarcinoma cells in response to increasing doses of IR, with cells treated with 10 Gy of IR secreting approximately 15 times more active LOX enzyme than control cells (Figure 1A). The total amount of proteins in CM was only minimally changed and equal amounts of protein were loaded for SDS-PAGE and immunoblotting. The increase in LOX secretion was paralleled by an increase in LOX activity as determined in CM, derived from cells treated with increasing doses of IR (Figure 1B).

To exclude an unspecific leakage of proteins in CM due to IR-damaged cells, CM were also tested against several other secreted proteins. A secretion profile of multiple factors was obtained using antibody arrays. Known secretory factors such as PDGFA (platelet derived growth factor alpha) showed increased secretion in response to IR whereas THBS1 (Thrombospondin 1) levels decreased with IR in a dose dependent manner. Furthermore, LDH, a classic marker for cell leakage, was not increased in response to irradiation (Additional file 1: Figure S1). Thus, no general protein leakage could be observed, indicating specific, IR-mediated LOX secretion.

**Figure 2:**
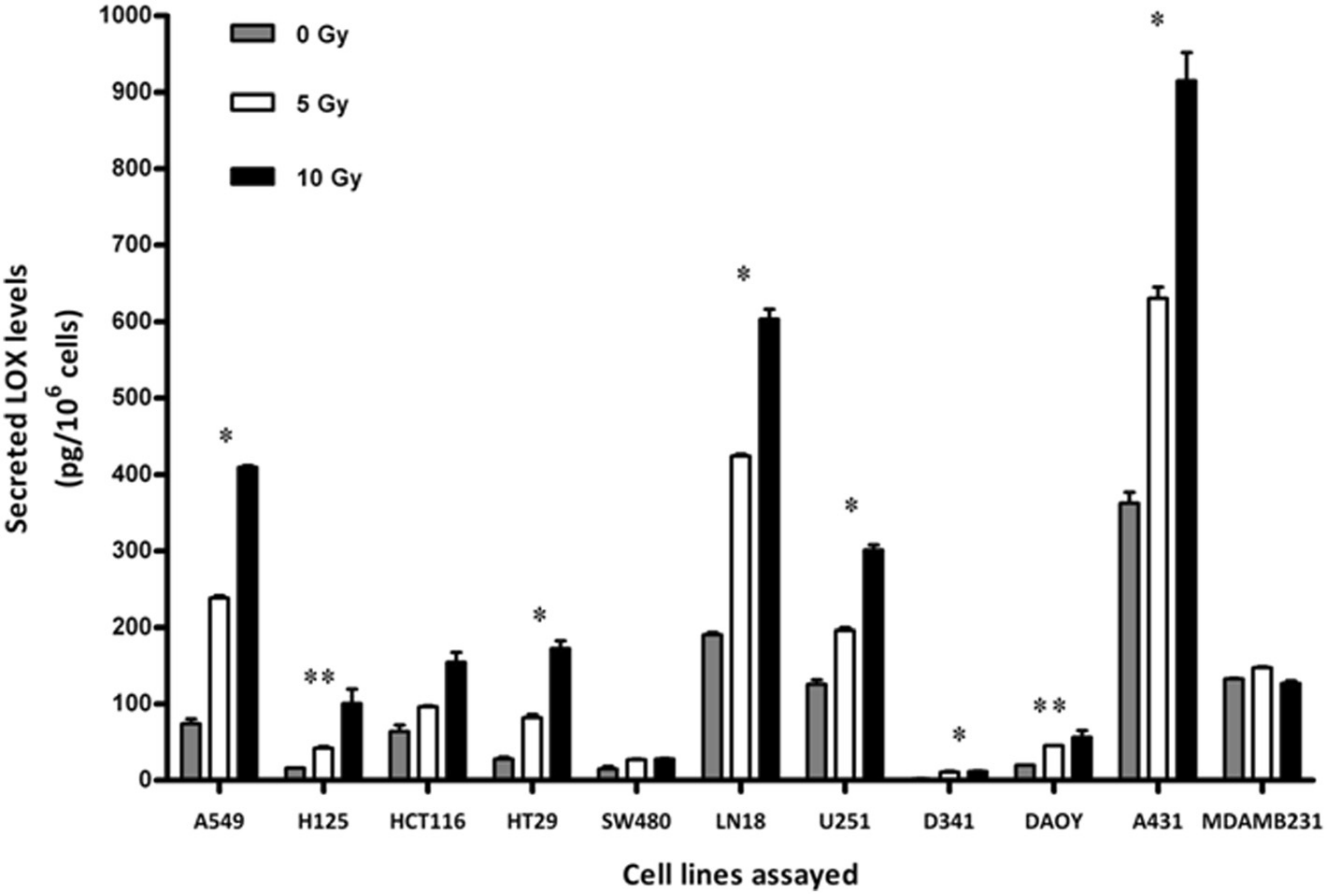
**LOX secretion in response to tumor cell irradiation from different tumor cell lines.** ELISA-based quantification of LOX in supernatants from irradiated tumor cell lines (*: significantly different for 5 and 10 Gy vs control samples, p < 0.05; **: significantly different only for 5 Gy vs control samples, p < 0.05).

To confirm IR-enhanced LOX secretion in additional cell lines, a LOX-directed ELISA was used to quantitatively detect the level of LOX in supernatants of irradiated tumor cells (0, 5, 10 Gy). With the exception of MDA-MB-231 cells, all tumor cell lines studied demonstrated enhanced LOX secretion in response to IR, but to different magnitudes depending on the cell type (Figure 2). To probe the level of secreted LOX over time, LOX was analyzed in CM of selected, irradiated cell lines at several time points after irradiation (10 Gy). A steady increase of secreted LOX, even though to different extents, could be determined in all three cell lines investigated (A549, SW620, A431) (Additional file 2: Figure S2). These results suggest that several tumor cell types exhibit increased LOX secretion in response to IR as a mode of stress response but that each tumor cell type may respond differently to varying doses of IR.

### Patupilone inhibits hypoxia-induced but not IR-induced LOX secretion

**Figure 3:**
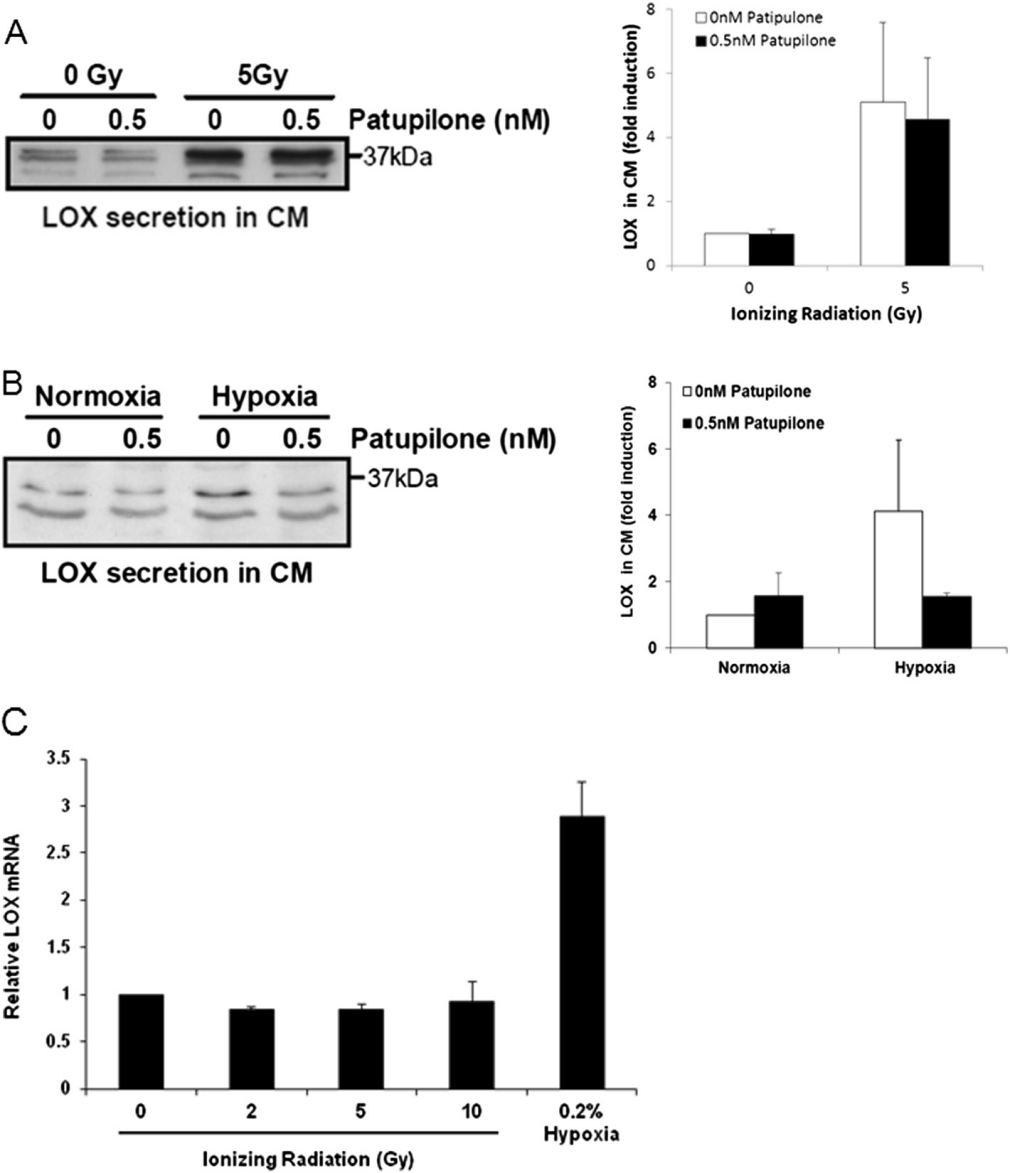
**Patupilone counteracts hypoxia- but not IR-induced LOX-secretion. (A)** Western blot of basal and IR-induced LOX in conditioned media derived from patupilone-untreated or patupilone-pretreated (0.5 nM) cells and quantification of band intensities from 3 independent experiments. **(B)** Western blot of basal and hypoxia-induced LOX in conditioned media derived from patupilone-untreated or patupilone-pretreated (0.5 nM) cells and quantification of band intensities from 3 independent experiments. **(C)** LOX gene transcription 16 h after irradiation and hypoxia, determined by RT-PCR, averaged over 3 independent experiments.

The microtubule stabilizing agent patupilone has previously been shown to abrogate hypoxia-induced protein expression, to sensitize for radiotherapy and to inhibit IR-induced VEGF and MMP secretion [11,34-38]. Thus, patupilone could potentially be used in combination with IR to abrogate any IR-induced LOX-related invasive or malignant phenotype. To investigate a putative counteracting effect of patupilone on IR-induced LOX-secretion, A459 tumor cells were pretreated with patupilone 24 hours prior to irradiation and LOX-secretion in CM was measured over 16-20 hours after irradiation. No change in basal or IR-induced LOX secretion was observed in CM derived from un-treated or pretreated cells with 0.5 nM patupilone, a dose previously shown to inhibit IR-induced VEGF and MMP-secretion in A549 cells (Figure 3A) [11,38]. These results suggest potentially different mechanisms governing LOX and VEGF or MMP secretion in response to IR.Even though patupilone did not affect IR-induced LOX secretion, we investigated hypoxia-induced LOX secretion to be regulated by patupilone. To this end, A549 tumor cells were pretreated with patupilone (0.5 nM) for 24 hours, followed by cell culture in normoxic and hypoxic (0.2% oxygen) conditions for 16-20 hours. Thereafter, conditioned media were collected to assess LOX secretion. As opposed to IR-induced LOX-secretion, hypoxia-induced LOX secretion was abrogated in patupilone-pretreated cells to the basal level as observed under normoxic conditions (Figure 3B). These results suggest that patupilone could potentially be used to inhibit LOX secretion from hypoxic tumors but that, at least at the concentration used here, patupilone might not prevent IR-induced LOX secretion. Furthermore, these results suggest differential mechanisms governing LOX secretion promoted by hypoxia and IR.

In contrast to previously investigated HIF-1-mediated LOX-secretion in response to hypoxia, IR-induced LOX secretion did not result from increased LOX-transcription in the A549 lung adenocarcinoma cells. While hypoxia strongly induced LOX gene expression, LOX-transcription remained constant in response to irradiation with increasing doses of IR as determined at the 16 h time point after irradiation (Figure 3C; see Additional file 3: Figure S3 for the SW620 colon carcinoma cell line). IR-induced LOX-transcription could not be observed at earlier or later time points after irradiation either (A549, SW620; Additional file 4: Figure S4). Thus, enhanced LOX secretion in response to irradiation does not result from increased transcription but rather from a post-transcriptional mechanism.

### IR-induced LOX promotes the invasive capacity of naïve tumor cells

**Figure 4:**
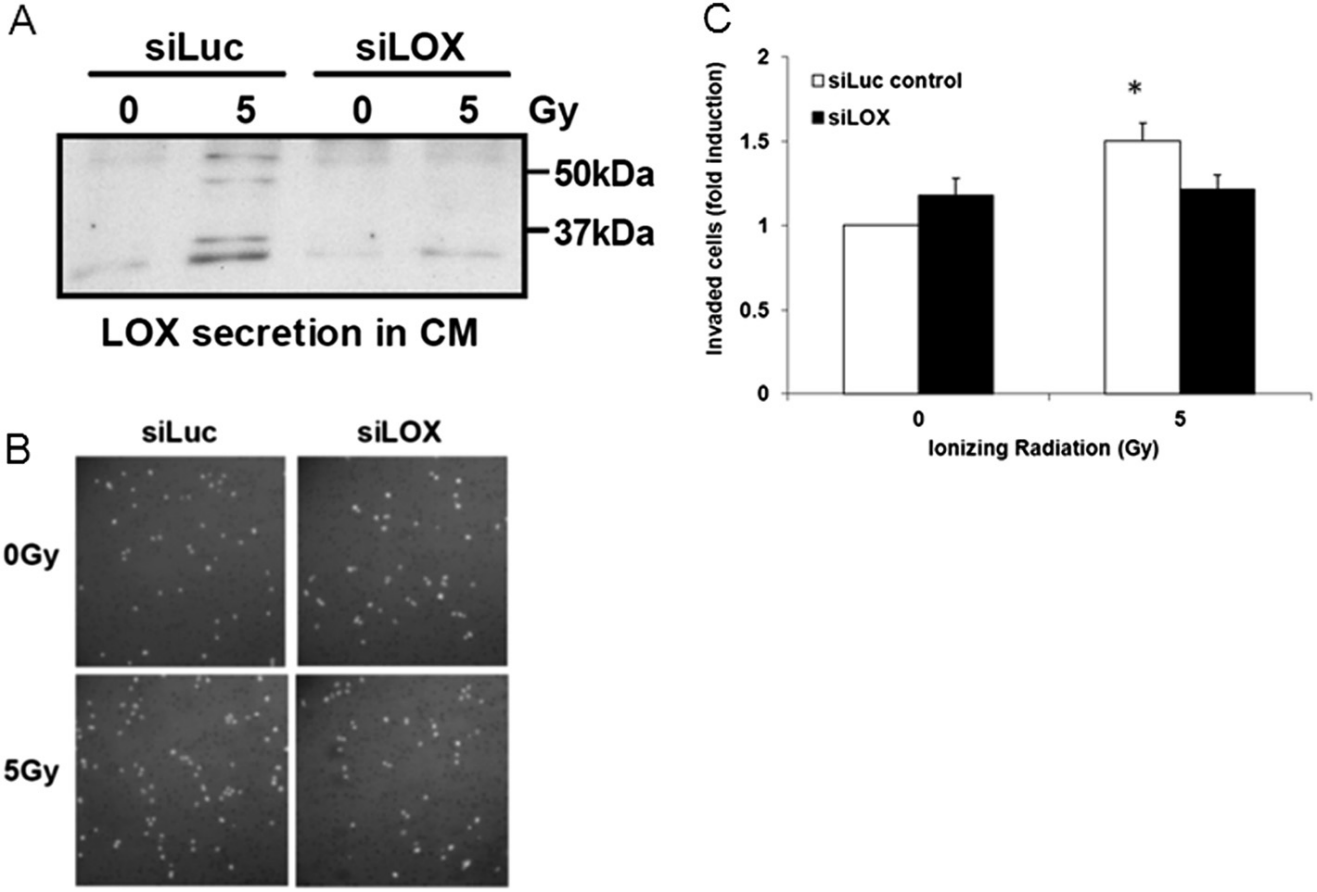
**IR-induced LOX promotes the invasive capacity of naïve tumor cells. (A)** Western blot of LOX in conditioned media derived from untreated and irradiated A549 cells, transfected with siLuc-control or siLOX-RNA. **(B)** Representative pictures of invaded A549 tumor cells stimulated with conditioned medium derived from un-irradiated and irradiated, siLuc-control- and siLOX-RNA transfected cells. **(C)** Quantification of invading A549 tumor cells stimulated with conditioned medium derived from un-irradiated and irradiated, siLuc-control- and siLOX-RNA transfected cells, averaged over 3 independent experiments.

LOX promotes cancer cell invasion and dissemination. To determine a functional role of IR-induced LOX secretion, we analyzed the invasive capacity of naïve A549 cells, stimulated with conditioned media derived from sham- or irradiated control and anti-LOX siRNA-targeted A549 tumor cells (Figure 4A). We observed a 50% increase in the number of invaded cells in the cell population stimulated with conditioned medium derived from irradiated, control-transfected cells when compared to sham-irradiated cells (Figure 4B-C). Interestingly, conditioned media derived from irradiated, LOX siRNA-transfected cells did not stimulate the invasive capacity of naïve A549 tumor cells, thereby demonstrating LOX-specificity. These results suggest that IR-induced LOX might promote an invasive tumor cell phenotype in naïve or sublethally irradiated cells.

### Irradiated tumor xenografts exhibit increased LOX secretion

**Figure 5:**
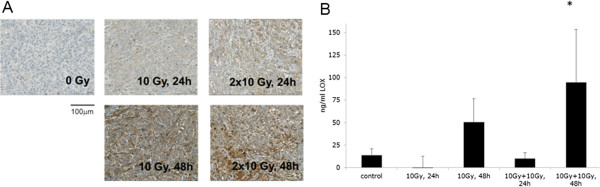
**IR-enhanced LOX in tumor xenografts. (A)** Tumor tissue from A549-derived tumor xenografts treated with 1 × 10 Gy and 2 × 10 Gy (with 12 h between fractions) and evaluated by immunohistochemistry for LOX at different time points after irradiation. Whole tumor sections were quantified for specific LOX-staining intensity. Each treatment group consists of 3 animals. At least 3 sections per tumor/animal were analyzed. **(B)** LOX in murine blood serum derived from mice with untreated or locally irradiated A549 tumor xenografts and quantified by ELISA at different time points after irradiation. Each treatment group consists of 3 animals.

Our studies demonstrated an increase of LOX secretion in irradiated tumor cell lines *in vitro*. To confirm these findings *in vivo*, we analyzed LOX secretion in A549-derived tumor xenografts in mice. Tumors were sham-irradiated, irradiated with a single dose of 10 Gy or irradiated with 2× 10 Gy, with the second dose applied 12 hours after the initial dose of IR. Tumors were then harvested for immunohistochemical analysis 24 and 48 hours after the single and last fraction of irradiation, respectively. We detected increased LOX-staining in tumor sections irradiated with both regimens of IR, with more enhanced LOX-staining at the 48 hour time point after irradiation (Figure 5A, see Additional file 5: Figure S5). Next, we also determined the level of human,tumor-derived LOX in the serum of tumor xenografted mice, which were irradiated with the two treatment regimens. We could not detect a change in the basal level of LOX 24 hours after irradiation. Interestingly though, an increased amount of LOX could specifically be detected in the serum of mice 48 hours after irradiation with 10 Gy. This level was even further increased in the serum of mice that were irradiated with the additional IR-fraction of 10 Gy (Figure 5B). Locoregional irradiation of mice not carrying tumor xenografts did not result in increased LOX in the serum above basal level and mouse-LOX levels did not change (data not shown). Thus, our results confirm that IR can also promote LOX secretion from tumors *in vivo.*

## Discussion

In this study, we demonstrated that LOX tumor cell secretion is promoted by IR *in vitro* and *in vivo* and that IR-induced LOX functionally promotes invasion of cancer cells *in vitro*. These findings suggest that LOX contributes to a coordinated stress response of cancer cells to IR. Along with increased secretion of VEGF, MMP, and TGF-β, among others [12-17], these responses may lead to cell survival, invasion, and dissemination of sublethally irradiated cancer cells and thereby contribute to locoregional and distant treatment failure.

LOX gene expression is induced by the activation of the primary transcription factor HIF-1α under hypoxia [23], but also in response to several cytokine-activated signal transduction cascades [23-25,39]. Eventually, this results in enhanced LOX levels secreted into the microenvironment. We here demonstrate that irradiation of tumor cells will also lead to increased amounts of secreted LOX, however in a gene transcription- and hypoxia-independent way. While hypoxia activates the transcriptional activity of HIF-1α and subsequently LOX-gene expression, IR did not activate HIF-1α or LOX-gene expression, at least in the cell systems investigated in this report (HIF-1α, data not shown). Elevated LOX levels could be identified in response to irradiation in conditioned media derived from established tumor cells from multiple different tumor entities and to different magnitudes. But so far we have not identified a correlation between IR-induced LOX levels and radiosensitivity in the cell lines investigated.

Thus, irradiation and hypoxia most probably regulate LOX via differential pathways, but the mechanism of IR-induced LOX-secretion remains elusive. TGF-β1 regulates LOX activity in osteoblastic cells on the pre-and posttranslational level [40], so IR-induced TGF-β1 could play a role in promoting LOX secretion from our tumor cells. Other possible mechanisms could involve IR-stimulated PI3K/Akt/mTOR signaling, a known stress response pathway to promote LOX protein synthesis. However, we could not detect IR-enhanced LOX-transcription. We exclude that LOX unspecifically leaked out from IR-damaged cell membranes since the total protein amount and other specifically investigated proteins were not upregulated after irradiation at the time point of CM-harvesting. Furthermore, LDH, a classic marker for cell leakage, was not increased in response to IR. Regardless of its mechanism, it will be important to investigate the functional effect of IR-enhanced LOX levels in the blood on tumor cell dissemination and premetastatic niches [27]. Detailed analysis of dose- and time dependence will be part of upcoming experiments investigating the relevance of IR-induced LOX secretion *in vivo*. In a tumor-unrelated *in vivo* model of pulmonary fibrosis, we recently observed that LOX expression is increased in fibrotic lungs induced by both irradiation and bleomycin [41]. Irradiation of lung tissue created LOX-dependent collagen crosslinking and subsequent lung fibrosis with a growth-permissive fibrotic microenvironment supporting metastatic growth. Our new results now demonstrate that IR also induces LOX secretion from tumor cells and in the tumor which is part of an early response and might not affect distant fibrogenesis, but might induce a bystander effect, as suggested by our in vitro data, on sublethally-irradiated or even distant unirradiated tumor cells. Of note, we here did not observe an increase of murine LOX in the serum of mice not carrying tumor xenografts but still locoregionally irradiated (data not shown). Furthermore, based on the detection of LOX in the serum of mice bearing irradiated tumor xenografts, future studies should also investigate LOX levels in human patients undergoing radiotherapy treatment.

Previous studies identified increased expression and secretion of LOX from tumor cells exposed to physiological levels of hypoxia as well as association of LOX with metastasis and poor survival in mammary carcinoma and head and neck cancer patients. Furthermore, inhibition of LOX activity eliminated tumor dissemination in an orthotopic mammary tumor model. Therefore, anticancer agents that concomitantly downregulate LOX might thereby also reduce tumor dissemination of sublethally treated tumor cells [27,28,30]. As such, epothilones including the clinically relevant compound patupilone, might be interesting candidates. While our study did not find a role for the microtubule stabilizing agent patupilone in inhibiting IR-induced LOX, patupilone did reduce hypoxia-induced LOX secretion. Of interest, we previously demonstrated that patupilone is as potent under normoxic as under hypoxic conditions and strongly sensitizes for ionizing radiation in particular *in vivo*. This coincides with interference of patupilone with the HIF-transcriptome, previously demonstrated for hypoxia-induced VEGF secretion [38]. Thus, the anti-metastatic effect of patupilone and related epothilone-derivatives, as characterized in other studies, might in part be due to reduced LOX secretion under hypoxia in patupilone-treated xenografts [42,43].

## Conclusion

Our results demonstrate to our best knowledge for the first time that LOX is secreted from tumor cells also in response to irradiation and indicate a differential regulation of LOX-expression and secretion in response to IR and hypoxia. These results suggest that LOX may contribute towards an IR-induced migratory phenotype in sublethally-irradiated tumor cells and tumor progression.

## Abbreviations

IR: Ionizing radiation; LOX: Lysyl oxidase; HIF: Hypoxia inducible factor; VEGF: Vascular endothelial growth factor; MSA: Microtubule stabilizing agent; MMP: Matrix metalloproteinase; TGF: Transforming growth factor.

## Competing interests

The authors declare that they have no competing interests.

## Authors’ contributions

Conception and design of the study: CS, JTE, MP. Data acquisition: CS, ABT, AS, VV. Data analysis and interpretation: CS, ABT, AS. Manuscript writing: CS, ABT, MP. All authors read and approved the final manuscript.

## Pre-publication history

The pre-publication history for this paper can be accessed here:

http://www.biomedcentral.com/1471-2407/14/532/prepub

## Supplementary Material

Additional file 1: Figure S1 No unspecific leakage of proteins in response to irradiation. Human angiogenesis antibody array showing secretion of multiple factors in supernatants derived from naïve (sham-irradiated) and irradiated (5 Gy) human A549 cells. Conditioned media was harvested 24 hours after irradiation. **(B)** Bar graph showing the levels of PDGFA (platelet-derived growth factor alpha) and THBS1 (Thrombospondin 1) in conditioned media derived from sham-irradiated or irradiated A549 cells. Levels of secreted proteins were measured by ELISA. **(C)** LDH-release in response to increasing doses of irradiation in the supernatants of A549-treated cells 24 hours after irradiation. Click here for file

Additional file 2: Figure S2 LOX secretion in response to tumor cell irradiation in different tumor cell lines. ELISA-based quantification of LOX in supernatants from A549 **(A)** SW620 **(B)** and A431 **(C)** tumor cells. CMs (from unirradiated and irradiated cells) were collected at the indicated time points after irradiation (10 Gy). The ratio of LOX in the CMs derived from irradiated versus unirradiated cells are shown. (*: significantly different from control conditions (0 Gy, 0 hour time point)). Click here for file

Additional file 3: Figure S3 Hypoxia but not IR enhances LOX gene transcription in SW620 colon carcinoma cells. LOX gene transcription was determined by RT-PCR, 16 h after treatment with increasing doses of IR and hypoxia, averaged over 3 independent experiments. Click here for file

Additional file 4: Figure S4 Absence of IR-induced LOX gene transcription. LOX gene transcription was determined in A549 lung carcinoma and SW620 colon carcinoma at the indicated timepoints after irradiation with 10 Gy. IR-induced CDKN1A gene expression was used as positive control. LOX and CDKN1A gene expression were determined by RT-PCR and averaged over 3 independent experiments. Click here for file

Additional file 5: Figure S5 IR-enhanced LOX in tumor xenografts. Tumor tissue from A549-derived tumor xenografts treated with 1 × 10 Gy and 2 × 10 Gy (with 12 h between fractions) were evaluated by immunohistochemistry for LOX at different time points after irradiation. Whole tumor sections were quantified for specific LOX-staining intensity. Each treatment group consists of 3 animals and at least 3 sections per tumor were analyzed. Bars for each treatment group show mean of % of area with no, low, medium and intense staining. Click here for file
