# Supplementary figures and images for: Ionizing radiation induces tumor cell lysyl oxidase secretion
Source: BMC Cancer. 2014 Jul 22;14:532. doi: 10.1186/1471-2407-14-532 (PMC4223762; doi:10.1186/1471-2407-14-532)

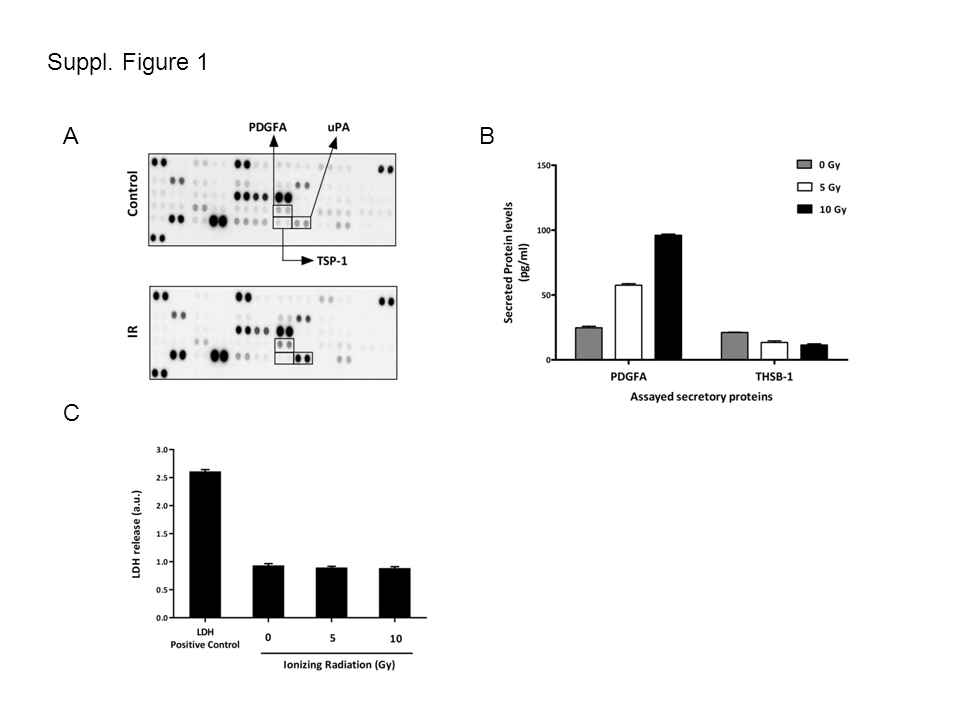

Supplement: Additional file 1: Figure S1 — No unspecific leakage of proteins in response to irradiation. Human angiogenesis antibody array showing secretion of multiple factors in supernatants derived from naïve (sham-irradiated) and irradiated (5 Gy) human A549 cells. Conditioned media was harvested 24 hours after irradiation. (B) Bar graph showing the levels of PDGFA (platelet-derived growth factor alpha) and THBS1 (Thrombospondin 1) in conditioned media derived from sham-irradiated or irradiated A549 cells. Levels of secreted proteins were measured by ELISA. (C) LDH-release in response to increasing doses of irradiation in the supernatants of A549-treated cells 24 hours after irradiation. [file 1471-2407-14-532-S1.tiff]

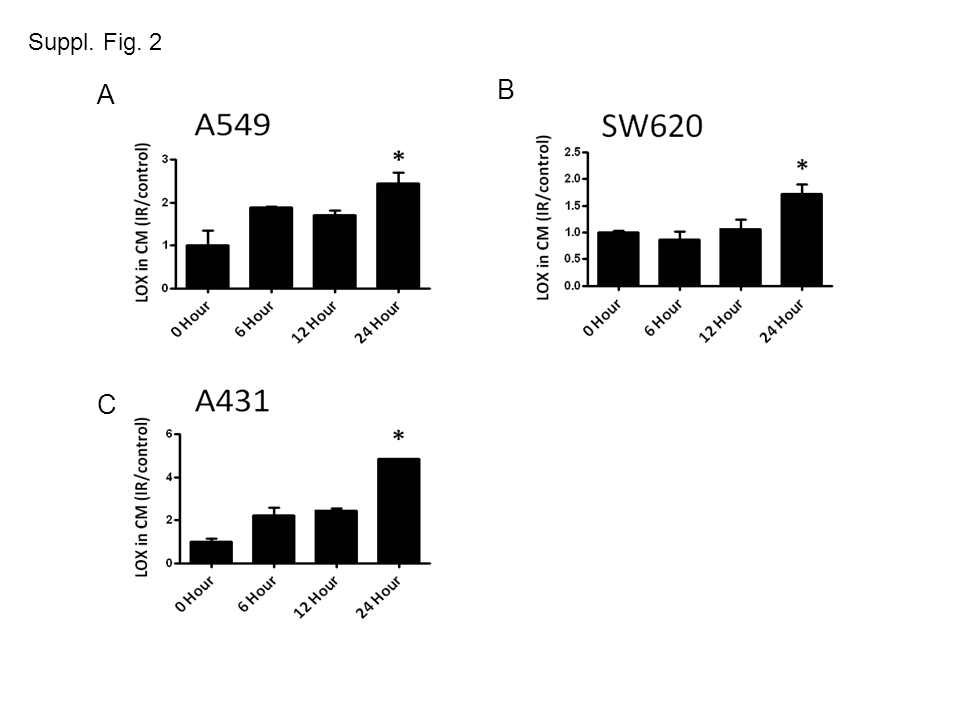

Supplement: Additional file 2: Figure S2 — LOX secretion in response to tumor cell irradiation in different tumor cell lines. ELISA-based quantification of LOX in supernatants from A549 (A) SW620 (B) and A431 (C) tumor cells. CMs (from unirradiated and irradiated cells) were collected at the indicated time points after irradiation (10 Gy). The ratio of LOX in the CMs derived from irradiated versus unirradiated cells are shown. (*: significantly different from control conditions (0 Gy, 0 hour time point)). [file 1471-2407-14-532-S2.tiff]

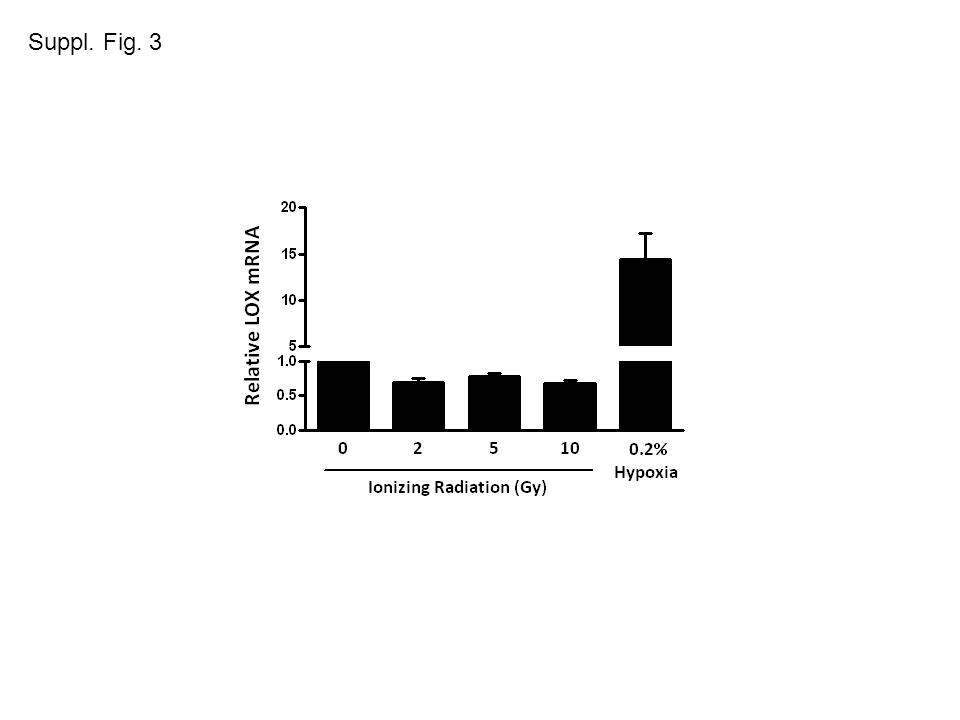

Supplement: Additional file 3: Figure S3 — Hypoxia but not IR enhances LOX gene transcription in SW620 colon carcinoma cells. LOX gene transcription was determined by RT-PCR, 16 h after treatment with increasing doses of IR and hypoxia, averaged over 3 independent experiments. [file 1471-2407-14-532-S3.tiff]

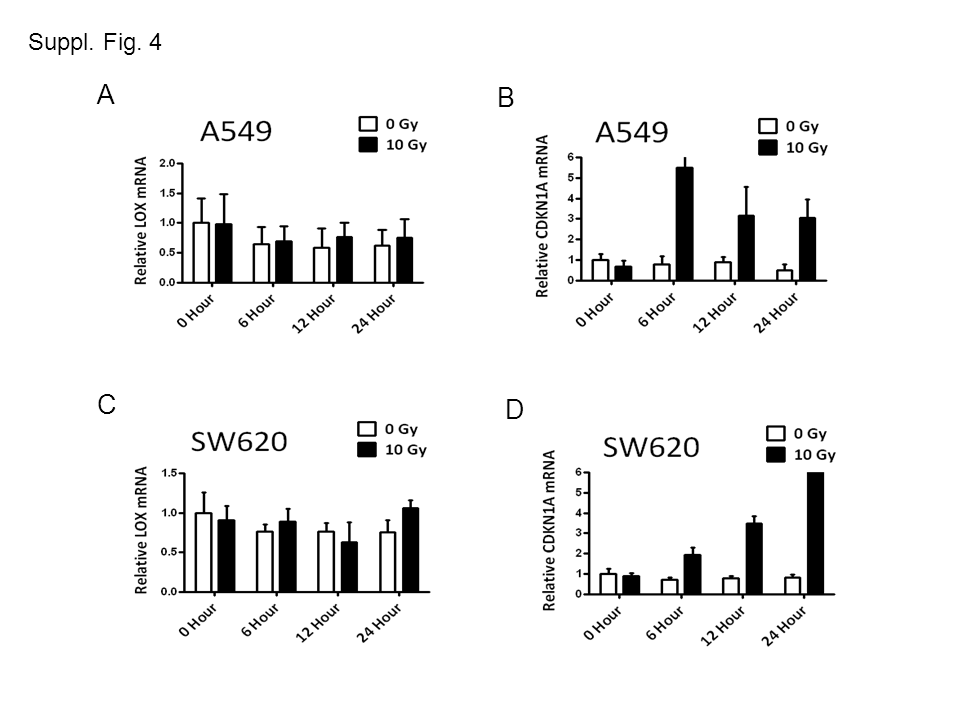

Supplement: Additional file 4: Figure S4 — Absence of IR-induced LOX gene transcription. LOX gene transcription was determined in A549 lung carcinoma and SW620 colon carcinoma at the indicated timepoints after irradiation with 10 Gy. IR-induced CDKN1A gene expression was used as positive control. LOX and CDKN1A gene expression were determined by RT-PCR and averaged over 3 independent experiments. [file 1471-2407-14-532-S4.tiff]

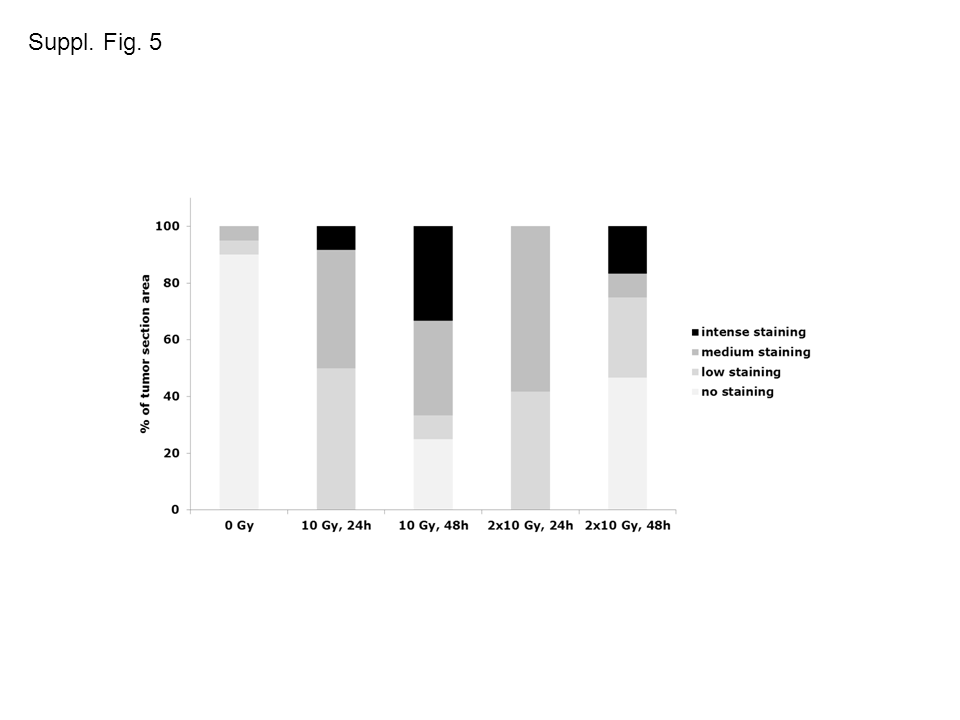

Supplement: Additional file 5: Figure S5 — IR-enhanced LOX in tumor xenografts. Tumor tissue from A549-derived tumor xenografts treated with 1 × 10 Gy and 2 × 10 Gy (with 12 h between fractions) were evaluated by immunohistochemistry for LOX at different time points after irradiation. Whole tumor sections were quantified for specific LOX-staining intensity. Each treatment group consists of 3 animals and at least 3 sections per tumor were analyzed. Bars for each treatment group show mean of % of area with no, low, medium and intense staining. [file 1471-2407-14-532-S5.tiff]
